# Prion diseases are undercompulsory notification in Brazil: Surveillance of cases evaluated by biochemicaland/or genetic markers from 2005 to 2007

**DOI:** 10.1590/S1980-57642008DN10400004

**Published:** 2007

**Authors:** Vilma Regina Martins, Hélio Rodrigues Gomes, Leila Chimelli, Sergio Rosemberg, Michele Christine Landemberger

**Affiliations:** 1 Ludwig Institute for Cancer Research, São Paulo, Brazil.; 2 Center for Research in Neurology (LIM/15), Faculty of Medicine of the University of São Paulo.; 3 Department of Pathology, School of Medicine, Federal University of Rio de Janeiro.; 4 Department of Pathology, Faculty of Medicine of the University of Sao Paulo.

**Keywords:** prion, prion diseases, transmissible spongiform encephalopathy, Creutzfeldt-Jakob disease, genetic polymorphism, prion, doenças por prions, encefalopatias espongiformes transmissíveis, doença de Creutzfeldt-Jakob, polimorfismo genético

## Abstract

The emergence of the new variant of Creutzfeldt-Jakob disease (vCJD) in the United Kingdom has raised concerns over the risks of this prion disease in other parts of the world. Since 2005, human prion diseases have been under compulsory notification in Brazil. It is well known that some polymorphisms within the cellular prion gene (PRNP) have been associated to a higher susceptibility to sporadic CJD (sCJD) and vCJD.

**Objectives:**

To describe the first notified cases and to evaluate the presence of mutations and polymorphisms of the PRNP in these cases.

**Methods:**

Thirty-five notified cases were evaluated by clinical, auxiliary exams and biochemical and/or genetic tests and classified according to the World Health Organization criteria for CJD. A control group (N=202) was included for the purpose of comparing the genetic analyses.

**Results:**

Twenty seven cases (74%) were classified as possible sCJD while 51% fulfilled the criteria for probable sCJD. Brain tissue analysis was available in three cases, where two were classified as definite sCJD and one as unconfirmed sCJD. Mutation of the PRNP was not found, and regarding the codon 129 polymorphism, valine in both alleles (Val129Val) was more frequent in patients than in the control group (OR=4.98; 1.55-15.96; p=0.007) when all possible cases were included, but not when only probable cases were considered.

**Conclusions:**

Our data did not show correlation of PRNP polymorphisms with probable sCJD cases. It is necessary to work toward notification of all cases of possible CJD in Brazil and to increase the rate of definitive diagnoses.

## Abstract (pt)

O aparecimento da nova variante da doença de Creutzfeldt-Jakob (vDCJ) na Grã-Bretanha causou preocupações quanto aos riscos de doenças por príons em outras partes do globo. Desde 2005, doenças humanas por príons são de notificação compulsória no Brasil. É bem conhecido que alguns polimorfismos do gene da proteína príon celular (PRNP) têm sido associados a maior susceptibilidade a DCJ esporádica (DCJe) e a vDCJ.

**Objetivos:**

Descrever os primeiros casos notificados e avaliar a presença de mutações e polimorfismos do PRNP nesses casos.

**Métodos:**

35 casos notificados foram avaliados clinicamente, mediante exames complementares, testes bioquímicos e/ou genéticos e classificados de acordo com os critérios de DCJ da Organização Mundial de Saúde. Grupo controle (N=202) foi incluído para comparação dos dados da análise genética.

**Resultados:**

27 casos (74%) foram classificados como possível DCJe, dos quais 51% preencheram critérios para provável DCJe. Exame neuropatológico do encéfalo foi realizado em apenas 3 casos, dos quais 2 foram classificados como DCJe definida e um como DCJe não confirmada. Mutações do PRNP não foram encontradas e, com respeito ao polimorfismo do códon 129, valina em ambos os alelos (Val129Val) foi mais freqüente em pacientes do que em controles (OR=4,98; 1,55-15,96; p=0,007) quando todos os casos foram investigados, mas não quando apenas casos prováveis foram incluídos.

**Conclusões:**

Nossos dados não mostram correlação dos polimorfismos do PRNP com provável DCJe. É necessário ampliar a notificação de todos os casos de possível DCJ no Brasil e o diagnóstico definitivo.

The Transmissible Spongiform Encephalopathies (TSE) are rare and invariable fatal neurodegenerative diseases of both humans and animals. Prions, the causative etiological agent of these disorders present unique biochemical characteristics. It is believed that they are devoid of nucleic acids and composed only by a single protein PrPSc. This protein is an abnormal conformational isoform of a cellular protein abundantly expressed in brain and very conserved among species, namely the cellular prion protein (PrPC).^1,2^

The “protein only hypothesis” stated by Stanley Prusiner in 1983 proposes that PrPSc interacts with PrPC and generates new infectious molecules by converting the latter’s structure.^3^ The fundamental role of PrPC as substrate for this conversion has been demonstrated when PrP^C^-null mice were bred and proved to be totally resistant to prion infection.^4^ In humans, TSE or prion diseases can be transmitted as inherited or acquired forms although sporadic spontaneous onset of the disorder has also been proposed. Genetic Creutzfeldt-Jakob Disease (CJD), Gerstmann-Straeussler-Scheinker syndrome (GSS) and Fatal Familial Insomnia (FFI) are inherited forms of prion disease caused by specific mutations or insertions in the cellular prion protein gene (*PRNP*).^5^ Iatrogenic transmission of prion diseases have been reported after treatment of patients with growth hormone purified from human pituitaries,^6^ contaminated deep brain electrodes,^7^ cornea transplantation;^8-10^ dura mater grafts;^11-13^ and brain surgery.^14^ The sporadic CJD (sCJD) is the most common human TSE with a worldwide incidence of 1 in 1.5 per million/year.^15^ In the mid 1990s a new variant of the CJD, vCJD, was identified in the United Kingdom.^16,17^ Epidemiological and experimental data associated vCJD with the consumption of contaminated products derived from cattle infected with bovine spongiform encephalopathy (BSE) or “mad cow disease”.^18^ Although it is believed that a large number of individuals have been exposed to these products, to date around 200 cases of vCJD have been identified in Europe, United States of America, Canada, Saudi Arabia and Japan.^19^ Thus, indicating that an interspecies barrier besides individual susceptibility are crucial determinants for disease transmission.^20^ The initial concerns over human to human transmission were corroborated by recent data showing that at least three patients with confirmed vCJD were probably contaminated by blood transfusion.^21-25^

Since no diagnostic test is available for routine prion screening in blood, transfusion of blood derivatives represent a further iatrogenic risk of TSE transmission among humans. In this context, surveillance systems for prion diseases have been established in a large Collaborative Study Group (EUROCJD) which includes Australia, Austria, Canada, France, Germany, Italy, the Netherlands, Slovakia, Spain, Switzerland and the UK. At a later date, this group also incorporated the Extended European Collaborative Study Group of CJD (NEUROCJD) integrating Belgium, Denmark, Finland, Greece, Iceland, Ireland, Israel, Norway and Portugal. Both projects are co-ordinated from the National CJD Surveillance Unit based in Edinburgh, Scotland (for more information see http://www.eurocjd.ed.ac.uk). In the USA, the National Prion Disease Pathology Surveillance Center (NPDPSC) was established at the Division of Neuropathology of Case Western Reserve University National (for more information see http://www.cjdsurveillance.com). Argentina also has an organized national surveillance system, The Neuropathology and Molecular Biology of Transmissible Spongiform Encephalopathies Referral Center at the Neuropathology Laboratory Raúl Carrea Neurological Research Institute, FLENI (for more information see http://www.fleni.org.ar/).

The clinical diagnosis of prion disease is based on specific clinical signs and symptoms, electroencephalography (EEG), magnetic resonance images (MRI), analysis of 14.3.3 protein in cerebrospinal fluid (CSF), brain biopsy, autopsy and immunoassays for prion protein associated with spongiform degeneration. According to the criteria of the World Health Organization, WHO (see http://www.who.int/entity/zoonoses/diseases/Creutzfeldt.pdf) a possible sCJD is defined as a progressive dementia with a duration of less than two years, atypical EEG or not performed and at least two out of the following clinical features: myoclonus, visual or cerebellar disturbance, pyramidal or extrapyramidal dysfunction or akinetic mutism. The classification of a probable case of CJD includes the parameters used for possible CJD plus typical EEG (generalized triphasic periodic complexes at approximately one per second) regardless of the clinical duration of the disease and/or a positive 14.3.3 assay in CSF. In order to confirm a definite sCJD, neuropathological evaluation (presence of spongiform encephalopathy and/or the presence of protease resistant prion protein) is mandatory. Although some clinical, MRI and neuropathological features differ between vCJD and sCJD, the definitive diagnosis for the former is also dependent on neuropathological criteria.

In 2001, the Brazilian Ministry of Health devised special actions aimed at reducing risks of CJD transmission within the country, and in July 2005 CJD was included amongst the diseases under mandatory notification and investigation.^26^ To date, 35 cases have been notified and analyzed by biochemical and genetic markers where 51% of these were classified as probable sCJD. Genetic forms of prion diseases due to recognized pathogenic *PRNP* mutations were not found. Neuropathological evaluation was feasible in three cases, two of which were classified as definite sCJD.

## Methods

### Patients and controls

The data presented in the present study were obtained from the notification forms of patients with possible CJD reported to the Sanitary Vigilance Secretariat (SVS) in 13 Brazilian States. The analyses comprise only notified patients whose CSF and/or blood samples were collected. A group of 202 healthy adults without any previous history of neurological disease, psychiatric disease or signs/symptoms suggestive of any type of spongiform encephalopathy was used as control for *PRNP* analysis. Informed consent was obtained from controls, patients, or persons legally responsible for them.

### Clinical parameters and WHO classification

Clinical diagnosis as well as EEG and MRI were performed by a physician at the region where the patient was located. Presence of periodic sharp wave complexes in EEG and abnormal high signal in the cortex, caudate nucleus, putamen and thalamus on FLAIR, T2 and/or diffusion-weighted in MRI were considered as suspicious. A notification form was filled out with all possible data while cerebrospinal fluid and blood samples were collected and sent to specialized centers following a pre standardized chronogram^26^ to evaluate 14.3.3 protein levels and for *PRNP* sequencing respectively.

According to the WHO criteria, a *possible* sCJD presents a rapidly progressive dementia, an atypical or not performed EEG and at least two of the clinical features: myoclonus, visual or cerebellar disturbance pyramidal or extrapyramidal dysfunctions or akinetic mutism. A *probable* sCJD presents all the parameters for possible sCJD plus typical EEG with diffuse background slowing and generalized periodic sharp wave complexes or the presence of the 14.3.3 protein in CSF. A neuropathological analysis showing the presence of spongiform degeneration and/or confirmation of protease-resistant prion protein by western blot or immunohistochemistry is mandatory for diagnosis of a definite sCJD.

The definition of possible vCJD requires a series of clinical parameters. One important clinical sign is an early and progressive psychiatric disorder (clinical duration >6 months) which must be accompanied of four of the following symptoms: early psychiatric symptoms (depression, anxiety, apathy, withdrawal, delusions), persistent painful sensory symptoms (pain and/or dysaesthesia), ataxia, chorea/dystonia or myoclonus and dementia. A probable vCJD diagnosis includes all the above clinical signs for possible vCJD plus a bilateral symmetrical pulvinar high signal on MRI brain scan and an atypical EEG.

A definite case of familial CJD is diagnosed when a pathogenic *PRNP* mutation is recognized or when any of these mutations are present in a first-degree relative.

### Quantification of 14.3.3in CSF

14.3.3 protein was detected in CSF by the immunoblotting technique at the Neurological Investigation Center (LIM 15) of the University of São Paulo School of Medicine as previously described.^27^ Briefly, a 15 µL CSF sample was submitted to electrophoresis in polyacrylamide gel with dodecyl sulfate and then transferred to a polyvinylidene difluoride (PVDF) membrane. Protein transfer was carried out in a Tris-glycine buffer to a PVDF membrane at 200mA over a 2-hour period. Membrane blocking was achieved using 5% skimmed milk and 0.05%Tween 20, in a saline/phosphate buffer. The membrane was incubated with a polyclonal antibody against an epitope in the N-terminal portion of the b-isoform of the human 14.3.3 protein, which reacts amply with members of the 14.3.3 protein family (sc 629; Santa Cruz Biotech, Santa Cruz, CA) at a 1:2,000 dilution. A 30 KDa band, corresponding to 14.3.3 protein, was detected by chemoluminescence (ECL; Amersham, Arlington Heights, IL). A high sensitivity X-ray film was exposed to the membrane. Positive and negative controls were included in all experiments.

### Analysis of *PRNP* sequence

Notified cases and healthy controls were evaluated for the *PRNP* by direct sequencing and/or denaturing high-performance liquid chromatography (DHPLC) as previously described.^28^ The experiments were conducted at the Molecular and Cellular Biology group at the Ludwig Institute for Cancer Research in São Paulo. DNA was extracted from a 3 mL aliquot of whole blood using the Wizard Genomic DNA Purification Kit® (Promega). Primers (IDT, SP, Brazil) were designed to amplify two different overlapping fragments of the *PRNP* open reading frame (ORF). Fragment 1 amplifies nucleotides 77 to 497 (421 bp) using primers Forward1: 5’ATG CTG GTT CTC TTTGTG 3’ and Reverse1: 5’AAC GGT CCT CAT AGT CAC TGC 3’. The cycling conditions were 95ºC for 5 min, followed by 35 cycles of 95ºC for 1 min, 64ºC for 1 min and 72ºC for 1 min, followed by a final extension of 72ºC for 10 min. Fragment 2 amplifies nucleotides 455 to 858 (404 bp) using Forward2: 5’ TCA TGG TGG TGG CTG GGG TCA 3’ and Reverse2: 5’ CGC CTC CCT CAA GCT GGA AAA 3’. The cycling conditions were 95ºC for 5 min, followed by 35 cycles of 95ºC for 1 min, 66ºC for 1 min and 72ºC for 1 min, followed by a final extension of 72ºC for 10 min. The PCR products were sequenced with the DYEnamic ET terminator sequencing kit (Amersham Pharmacia Biotech) according to manufacturer’s instructions using an ABI Prism–377 apparatus (Perkin-Elmer).

Denaturing high-performance liquid chromatography (DHPLC) of PCR-amplified products using forward and reverse primers for fragment 1 (described above) and the forward (5’ ATCATACATTTCGGCAGT 3’) and reverse (5’ CTCCCTCAAGCTGGAA AAAGA 3’) primers for the second half of the *PRNP* ORF (nucleotides 463 to 867) were employed. A DNASep column (Transgenomic, CA) was used and the parameters of gradient and flow rate adjusted by the Wavemaker system control software (Transgenomic).

### Pathology and immunohistochemistry assays

Paraffin blocks in which the brain tissue was embedded, were sent from various states for histological analysis. The block surface containing the specimen was decontaminated by immersion into 1% formic acid for one hour. Five µm sections were stained with hematoxylin-eosin. Brain sections were treated with 4M guanidine thiocyanate at 4ºC for 2 hours followed by immunohistochemistry with anti-PrP 3F4 (Abcam # ab10282) at 1:500 dilution).

### Statistical analysis

The frequency of *PRNP* polymorphisms in patients and controls was analyzed by Fisher’s exact test. Association between *PRNP* alleles and possible or probable CJD was measured by the Odds Ratio (OR) and respective 95% confidence interval (CI). The OR was estimated by unconditional logistic regression (SPSS program version 12.0.1 Chicago, IL). The level of significance was set at p <0.01.

## Results

**Figure 1:**
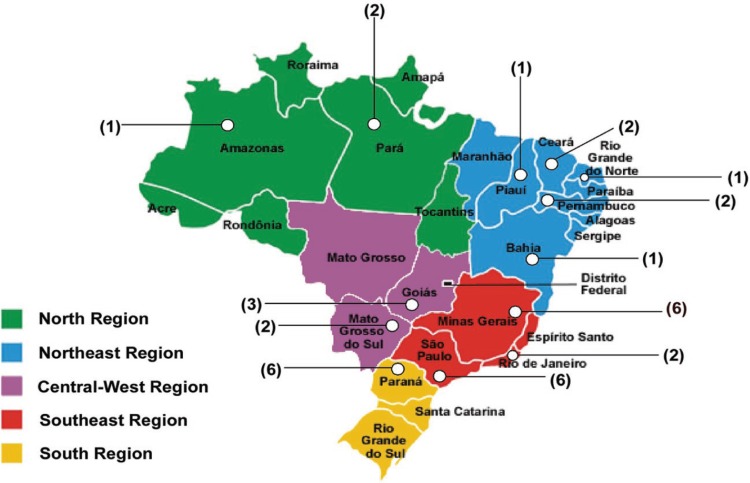
The notified cases for possible CJD evaluated by biochemical and/or genetic markers were distributed throughout the Brazilian territory. Numbers in brackets represent the quantity of reported patients in the state indicated.

From August 2005 through to September 2007, 35 suspected cases of CJD reported to the System of Sanitary Vigilance of the Ministry of Health (SVS-MS) were evaluated by biochemical and/or genetic tests. The mean age of the patients at notification was 58.2 years with a median of 62 years (range 22-81 years). Males accounted for 60% of the cases. Figure 1 shows the distribution of these cases around the country.

Clinical symptoms were noted down in the notification form at the date that cases were reported and complete data were available in 31 (89%) out of 35 cases. According to the WHO definitions, 26 (74%) of the notified patients (Table 1) were initially classified as possible sCJD (possible + probable). Table 2 shows that 31 out of 35 notified patients presented a rapidly progressive dementia (duration of symptoms shorter than two years) whereas in the other 4 patients the notification form was incomplete thereby preventing proper evaluation. The presence of at least two additional clinical signs or manifestations such as visual and cerebellar disorders, pyramidal and extrapyramidal signs or akinetic mutism was found in 26 patients. Thus, these last patients fulfilled the criteria for possible sCJD (Table 1). The most frequent symptoms were myoclonus (80%), pyramidal signs in 68%), ataxia (65%), cerebellar (55%) and visual disorders (48%), akinetic mutism (42%) and sleep alterations (48%) (Table 2).

**Table 1:** Classification of notified patients according to WHO criteria for human prion diseases.

Possible sCJD	Probable sCJD	Possible vCJD	Unclassified	
			Insufficient clinical signs	Incomplete form
n=8 (23%)	n=18 (51%)	n=2 (6%)	n=3 (9%)	n=4 (11%)

**Table 2:** Clinical signs presented in patients whose notification form was complete.

Clinical signs	n (%)
Progressive dementia (less than 2 years)	31 (100)
Myoclonus	25 (80)
Pyramidal dysfunction	21 (68)
Ataxia	20 (65)
Early Psychiatric symptoms	17 (55)
Cerebellar disturbances	17 (55)
Visual disturbance	15 (48)
Sleep disturbances	15 (48)
Extrapyramidal dysfunctions	14 (45)
Akinetic mutism	13 (42)
Persistent painful sensory symptoms (pain or dysaesthesia)	2 (7)

The WHO’s criteria to define a probable CJD include all requisites for possible sCJD plus the presence of 14.3.3 protein in cerebrospinal fluid (CSF) or typical EEG. The presence of the 14.3.3 protein in CSF implies a high diagnostic sensitivity and specificity of over 90% in sporadic cases of CJD.^29^ The protein levels were evaluated in 33 (94%) patients from the notified cases, 9 of which were positive (27%) (Table 3). Typical EEG is defined by diffuse background slowing and generalized periodic sharp wave complexes, which are found in at least two-thirds of all CJD cases.^30^ EEG was performed on 29 (83%) patients and presented a typical profile in 14 cases (49%) while an atypical appearance was observed in another 12 patients (42%). Three patients (9%) presented normal EEG (Table 3).

**Table 3:** Diagnostic approaches performed in the notified patients.

14.3.3 (n=33)			EEG (n=29)				MRI (n=25)			
Negative n (%)	Positive n (%)		Normal n (%)	Typical n (%)	Atypical n (%)		Normal w/o dif. n (%)	Normal w/ dif. n (%)	Typical w/ dif. n (%)	Other abnor. n (%)
n=24 (73%)	n=9 (27%)		n=3 (9%)	n=14 (49%)	n=12 (42%)		n=1 (4%)	n=4 (16%)	n=12 (48%)	n=8 (32%)

Brain scanning by magnetic resonance imaging (MRI) is also very useful although not included in sCJD definition by the WHO’s criteria. High signal abnormalities on T2 imaging in the cortex, putamen and caudate regions are indicative of classical sCJD.^31^ Of the 25 patients in whom MRI was conducted, typical signal abnormalities were observed in 12 (48%) (Table 3)

The analysis of clinical data, 14.3.3and EEG according to the WHO’s criteria showed that 51% of the notified patients fulfilled requisites for probable sCJD (Table 1).

It is very interesting to observe that 55% of the patients had early psychiatric symptoms while 7% presented persistent painful sensory symptoms (Table 2), which are more frequently observed in vCJD.^32,33^ Indeed, according to clinical and EEG results two of the notified patients fulfilled WHO’s criteria for possible vCJD (Table 1). Nonetheless, none of these patients presented bilateral symmetric pulvinar high signal on MRI brain scan which is a typical signal of vCJD.^34^ Thus, none of the patients fulfilled the WHO’s criteria for probable vCJD.

Mutations in *PRNP* have been associated to genetic forms of prion diseases. We sequenced the entire ORF (open reading frame) of *PRNP* in 27 (77%) of the 35 notified patients and no disease-specific *PRNP* mutations was found. Thus, none of these patients presented genetic forms of prion diseases. Additionally, the notification forms from 7 out of 8 patients where *PRNP* analysis was not conducted, reported having no first degree parent affected with a similar disease.

We also evaluated the presence of *PRNP* polymorphisms in these patients and compared them to a control group (Table 4). The deletion of one octarepeat domain in one allele was present in 7.4% (n=2) of the patients and in 5.0% (n=10) of the controls (p=0.160). The silent mutation at codon 117 presented in 4.5% of the control group (n=9) and found in 7.4% (n=2) of the notified patients (p=0.505).

**Table 4:** PRNP polymorphisms in notified cases compared to controls.

Residue Position	Genotype	Exposed (%)			Crude	
		Controls n=202 (%)	Possible CJD n=27 (%)		OR (95% CI)	p Value
Octarepeat	R12234 R12234/R1234 R1234	192 (95.0) 10 (5.0) 0 (0)	25 (92.6) 2 (7.4) 0 (0)		1.00 2.68 (0.68-10.59) NA	
117	Ala/Ala Ala/Ala_silent_	193 (95.5) 9 (4.5)	25 (92.6) 2 (7.4)		1.00 1.72 (0.35-8.40)	
129	Met/Met Met/Val Val/Val	112 (55.4) 81 (40.1) 9 (4.5)	15 (55.6) 6 (22.2) 6 (22.2)		1.00 0.56 (0.21-1.45) 4.98 (1.55-15.96)	

Although the genetic variants at codon 129 are not directly linked to any prion disease, the presence of methionine in homozygosis or heterozygosis has been associated to a higher susceptibility to sCJD, iatrogenic acquired and vCJD.^35,36^ The haplotype Met129Met (homozygous for methionine) was present in 55.6% (n=15) of the notified cases and in 55.4 (n=112) of the controls. Methionine in one allele (Metl129Val) was present in 40.1% of the controls (n=81) and in 22.2% (n=6) of the patients (p=0.241). Interestingly, valine in both alleles (Val129Val) was more frequent in patients than in the control group (OR=4.98 (1.55-^15^.96), p=0.007) (Table 4).

In order to evaluate the frequencies of these polymorphisms applying a more rigorous diagnostic criterion we compared the group classified as probable CJD with the control group (Table 5) or the group classified as probable CJD to the rest of the notified patients not fulfilling the criteria for probable sCJD (Table 6). No differences were found among these groups.

**Table 5:** PRNP polymorphisms in probable sCJD cases compared to the controls.

Residue Position	Genotype	Exposed (%)			Crude	
		Controls n=202 (%)	Probable sCJD n=15(%)		OR (95% CI)	p Value^a^
Octarepeat	R12234 R12234/R1234 R1234	192 (95.0) 10 (5.0) 0 (0)	14 (93.3) 1 (6.7) 0 (0)		1.00 1.37 (0.16-11.49) NA	
117	Ala/Ala Ala/Ala_silent_	193 (95.5) 9 (4.5)	14 (93.3) 1 (6.7)		1.00 1.53 (0.18-12.95)	
129	Met/Met Met/Val Val/Val	112 (55.4) 81 (40.1) 9 (4.5)	11 (73.3) 2 (13.3) 2 (13.3)		1.00 0.25 (0.05-1.17) 2.26 (0.43-11.81)	

**Table 6:** PRNP polymorphisms in probable sCJD cases compared to the other notified cases.

Residue Position	Genotype	Exposed (%)			Crude	
		Probable s CJD n=15 (%)	Other notified n=12 (%)		OR (95% CI)	p Value^a^
Octarepeat	R12234 R12234/R1234 R1234	14 (93.3) 1 (6.7) 0 (0)	11 (91.7) 1 (8.3) 0 (0)		1.00 1.27 (0.07-22-72) NA	
117	Ala/Ala Ala/Ala_silent_	14 (93.3) 1 (6.7)	11 (91.7) 1 (8.3)		1.00 1.27 (0.07-22.72)	
129	Met/Met Met/Val Val/Val	11 (73.3) 2 (13.3) 2 (13.3)	5 (41.7) 4 (33.3) 3 (25.0)		1.00 4.40 (060-32.50) 3.30 (0.41-26.37)	

The brain tissue was available for neuropathological diagnosis in three cases of the notified patients. In one such case the patient was classified as probable sCJD according to the WHO’s criteria but the presence of spongiform encephalopathy and presence of prion protein was not confirmed. The second case classified as probable CJD was confirmed as a definite sCJD. The last also confirmed for definite CJD, was not classified initially as possible CJD because it did not present clinical signs that fulfilled criteria for possible CJD in spite of a positive test for 14.3.3.

## Discussion

The Sanitary Vigilance group of each state has a key role in helping clinicians and making them aware about the compulsory notification of any possible human prion disease. It is important to observe that independent of geographic location of the state (Figure 1) most had notified patients notified and in the majority of the cases biological material (blood and CSF) arrived at the reference centers in São Paulo in an adequate condition.

Table 7 shows that clinicians and Vigilance Centers efficiently collected and adequately completed the notification form. The present data confirm that there was no over notification, at least when the material arrived for biochemical and genetic analysis. Indeed, 26 (74%) out of 35 patients fulfilled WHO’s criteria for possible sCJD and 2 (6%) fulfilled criteria for possible vCJD which are also criteria for compulsory disease notification. It is important to note that criteria for possible CJD are clinical and these data were missing in the notification forms of 4 patients. Thus, the actual number of patients classified as possible CJD could have been even higher.

**Table 7:** Data available in the notification form at the time 14.3.3 and/or PRNP analysis were requested (distribution by Brazilian States where cases were reported).

State	Total # cases	# Complete clinical data	# EEG	# MRI
AM	1	1	0	0
BA	1	1	1	1
CE	2	2	2	2
GO	3	3	3	2
MG	6	6	5	5
MS	2	2	2	1
PA	2	1	0	1
PE	2	2	2	2
PI	1	1	1	1
PR	6	5	5	1
RJ	2	2	2	2
RN	1	1	1	1
SP	6	4	5	5
Total	35	31	29	24

AM: Amazonas; BA: Bahia; CE: Ceará; GO: Goiás; MG: Minas Gerais; MS: Mato Grosso do Sul; PA: Pará; PE: Pernambuco; PI: Piauí; PR: Paraná; RJ: Rio de Janeiro; RN: Rio Grande do Norte; SP: São Paulo.

The 14.3.3 protein has been described in 90% of the patients with sCJD.^29^ Although we do not have definite CJD diagnosis, 57% of the notified cases fulfilled criteria for probable sCJD but only 44% of these were positive for 14.3.3. Additionally, a higher frequency of M129M polymorphism has been described in sCJD cases.^35,36^ Conversely, our data demonstrated that V19V was more frequent in notified cases than in control individuals while this result did not remain when probable sCJD cases were compared to controls. Indeed, the small number of patients in our analysis and more importantly, the lack of confirmation of definite CJD cases may have contributed to these discrepancies.

It is notable that data from EEG or MRI were available for patients from different regions in the country showing that these methods may not represent a technical limitation at least in the states where the disease was notified (Table 7). Nonetheless, the clinically poor condition of such patients might represent a constraint when needing transfer to a center offering these techniques. On the other hand, collecting CSF and peripheral blood in the regional hospital where the patient is located is more feasible.

We should emphasize the data showing that 2 patients fulfilled the classification of possible vCJD. Their MRI results do not permit inclusion as probable vCJD, however in these cases a tonsil biopsy would be of interest^37^ (see also http://www.who.int/entity/zoonoses/diseases/Creutzfeldt.pdf).

Finally, we have to seriously address the surprisingly low number of cases where brain tissue was available for definite neuropathological diagnosis. Many limiting factors could have contributed to this problem: loss of contact between sanitary vigilance and patients, carelessness of physicians and family members, non-compulsory necropsy, the low number of professionals trained to carry out necropsy in suspected patients, the limited number of centers safely equipped to perform necropsy and finally prejudice against these diseases.

In fact, neuropathological diagnosis of these diseases is the limiting factor to diagnosing prion diseases in Brazil and there efficient conduct should be adopted if we truly desire to ascertain the incidence of TSEs in the country.
